# Trophic isotopic carbon variation increases with pond’s hydroperiod: Evidence from an Austral ephemeral ecosystem

**DOI:** 10.1038/s41598-017-08026-6

**Published:** 2017-08-08

**Authors:** Tatenda Dalu, Ryan J. Wasserman, P. William Froneman, Olaf L. F. Weyl

**Affiliations:** 1 Zoology and Entomology, Rhodes University, P O Box 94, Grahamstown, 6140 South Africa; 2 School of Science, Monash University Malaysia, Jalan Lagoon Selatan, 47500 Bandar Sunway, Selangor Darul Ehsan Malaysia; 3 South African Institute for Aquatic Biodiversity, P Bag 1015, Grahamstown, 6140 South Africa

## Abstract

Trophic variation in food web structure occurs among and within ecosystems. The magnitude of variation, however, differs from system to system. In ephemeral pond ecosystems, temporal dynamics are relatively more important than in many systems given that hydroperiod is the ultimate factor determining the presence of an aquatic state. Here, using stable isotopes we tested for changes in trophic chain length and shape over time in these dynamic aquatic ecosystems. We found that lower and intermediate trophic level structure increased over time. We discuss these findings within the context of temporal environmental stability. The dynamic nature of these ephemeral systems seems to be conducive to greater levels of intermediate and lower trophic level diversity, with omnivorous traits likely being advantageous.

## Introduction

The study of trophic structure and community dynamics is a tenet of ecology and is considered central to our understanding of community ecology^1, 2^. Food webs are essentially species networks linked by trophic interactions that describe the ecosystem biodiversity and feeding relations^3^. These complex interactions are often figuratively depicted by nodes, which represent contributing biota (e.g. species) and connecting lines that represent interaction strengths between nodes. This allows for the characterisation of food webs using shape profiles and associated analyses. As such, food web height and shape have been identified as potentially important characterisations of the trophic structure. Thus, the overall dimensions of a given food web can provide insight into how ecosystems function and change in space and time^4, 5^.

It has been shown that the number of levels in energy transfer from primary producers to top predators is likely to be determined by ecosystem ambient temperature, habitat heterogeneity, productivity, size, and species richness^6, 7^. In addition, since the “height” of a food web is driven by nitrogen and the “width” largely facilitated by carbon, food web shape is also likely influenced by the availability of these elemental compounds^5, 6^. There has been much work done in this regard on permanent water bodies, whereby C and N availability is an aspect of resource accumulation, exploitation and standing biomass, facilitating longer or shorter food webs of various widths^5, 7–9^. Relatively little work has, however, been conducted on ephemeral water bodies.

Much work on the dynamic nature of food webs has been conducted^1, 2, 5, 10, 11^ and, it is now well recognised that trophic variation occurs among and within ecosystems across spatial and temporal scales^5^. In ephemeral pond ecosystems, temporal dynamics are particularly important given that hydroperiod is the ultimate factor determining whether the aquatic state is even present. Physico-chemical conditions are highly variable over the hydroperiod and likely have implications for succession patterns, particularly for internal community development. With regard to top-down processes in these ecosystems, much of the predation pressure in ephemeral ponds is imported over time through the arrival of organisms such as insects and amphibians that exploit these environments as profitable food patches^12, 13^. Thus, the relative importance of bottom-up and top-down pressures is likely to shift over time, and have significant implications for food web structure and ultimately, trophic shape. Here, we test for the changes in trophic chain length and shape in an ephemeral pond over time.

Ephemeral freshwater systems are widely distributed and include both lentic and lotic habitats^14^. These environments typically only exist as aquatic environments for limited periods and typically only hold water for a few days to months^8, 15–18^. Given their impermanency as an aquatic ecosystem, processes of basal resource accumulation, community development and food web complexity are fundamentally different to those of permanent water bodies^8, 19^. In lentic ephemeral ecosystems, communities are comprised of both internal recruits, in the form of biota emerging from dormant eggs, and external recruits such as insects and amphibians which are mobile and can therefore, invade from other habitats^12, 20^. The hydroperiod is a major factor determining community dynamics given that internal and external recruitment is often characterised by varying degrees of temporal separation, linked to hydroperiod state^13, 19–23^. For example, many of the internal recruits hatch early in an ephemeral ponds hydroperiod, while immigrating predator arrival occurs later^19, 24, 25^. The relative contribution and biomass of, and trophic exchange between these biotic components will, therefore, likely change over the course of the hydroperiod^12, 26^.

In this study, community food web metrics were used to assess the temporal changes in food web structure in a temperate ephemeral pond. To reveal variations in food web length and shape, we analysed community-wide metrics^27^ using stable isotopes (δ^15^N, δ^13^C) of invertebrate communities over the course of the pond’s hydroperiod i.e. five months. We were particularly interested in temporal changes in the shape of the isotopic space by conducting food web studies from a pond environment over four discrete points in time over the hydroperiod. We postulated that ephemeral pond food webs would change over time and that internal processes would be less important than the immigration of predators in contributing to changes in shape of the food web. We proposed that these environments would be characterised by: 1) simple, short food webs comprised of lower and intermediate trophic levels early on; 2) little change in the lower and intermediate trophic structure over time as expected based on trophic position i.e. δ^15^N values; and 3) an increases in food web length over time based on isotopic nitrogen and organic matter i.e. δ^13^C values.

## Results

**Figure 1:**
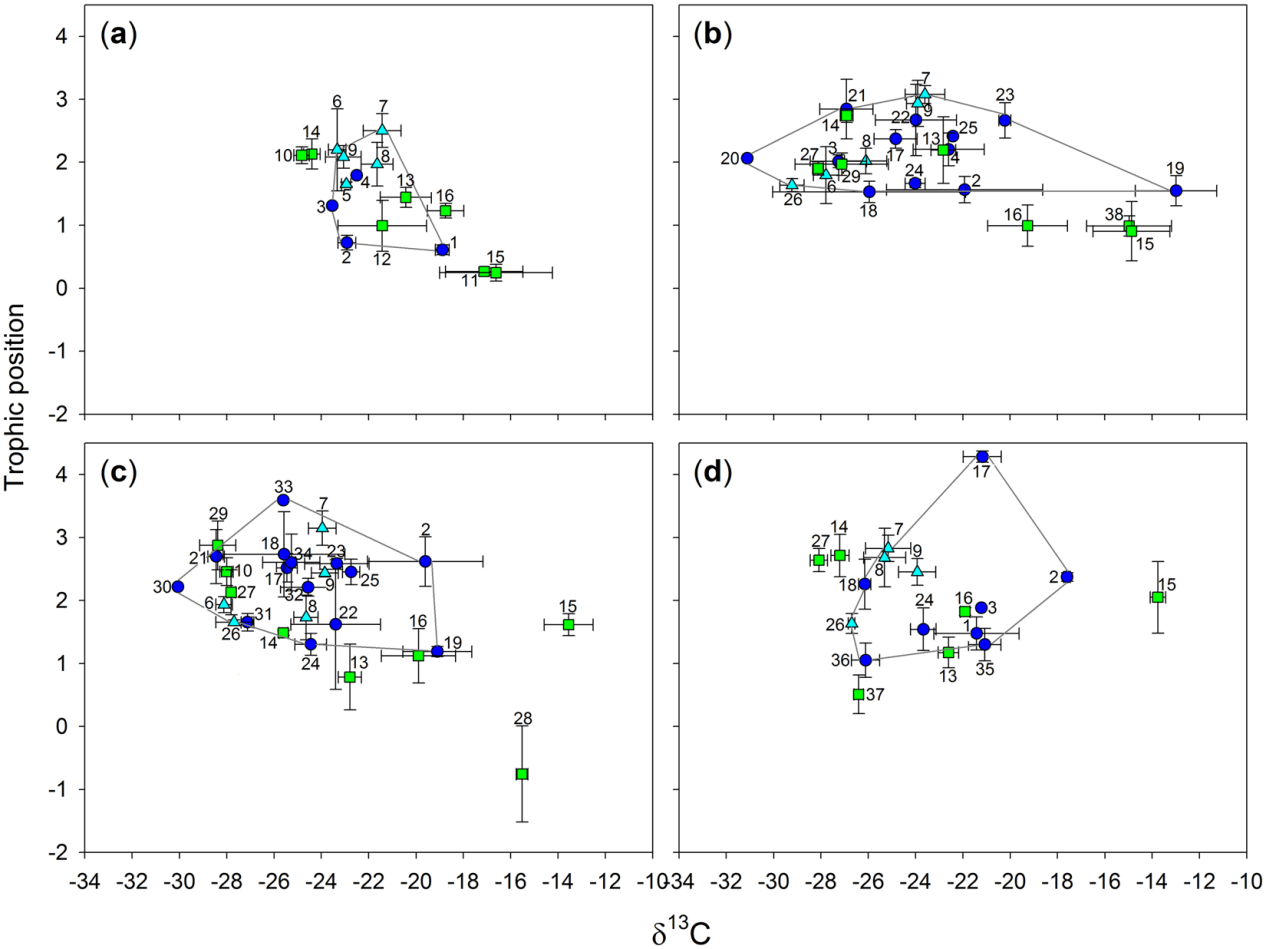
Stable isotope-based biplots of trophic position inferred from δ^15^N *vs* δ^13^C values over the course of a hydroperiod: (**a**) survey 1, (**b**) survey 2, (**c**) survey 3, and (**d**) survey 4 showing the convex hull areas encompassing all invertebrate taxa. For each sample point, *n* = 2–17 individuals and error bars represent ± standard deviations, green squares – plants (i.e. macrophytes, POM, detritus), dark blue circles – macroinvertebrates, light blue triangles – zooplankton. Taxa names for invertebrates see Table 1, 10 – *Cyperus marginatus*, 11 – detritus, 12 – filamentous algae, 13 – POM, 14 – *Marsilea* spp., 15 – *Sporobolus africanus*, 16 – sediment, 27 – *Crinium* spp., 28 – Euglenophyta, 29 – *Persicaria* spp., 37 – *Laurembergia repens* subsp. *brachypoda*, 38 – horse dung.

The invertebrate taxon richness increased from 9 during the first survey to 19 at survey 3 before decreasing to 13 during the final survey (Fig. 1; Table 1). Macroinvertebrates dominated during surveys 2 and 3 contributing to increased taxa richness, before decreasing at survey 4. During survey 1, the mean δ^13^C values of the invertebrates ranged from −18.9‰ (*Cypricercus* sp. 1) to −23.3‰ (*Daphnia magna*), while *Lovenula raynerae* having the highest trophic position (TP – 2.5) among the invertebrates analysed and *Streptocephalus* sp. (TP –1.3) having the lowest. During surveys 2 and 3, the δ^13^C values of the invertebrates ranged from −21.9‰ to −31.1‰ and from −19.1‰ to −30.1‰, respectively, with Chlorolestidae and *Baetis* sp. being δ^13^C depleted invertebrates during surveys 2 (−31.1‰) and 3 (−30.1‰), respectively (Fig. 1; Table 1). *Lovenula raynerae, Enithares sobria* and *Aeshna* sp. were the top predators, with TPs values of 3.1, 3.6 and 4.3 during surveys 2, 3 and 4, respectively. Macroinvertebrates were more enriched than the zooplankton (Fig. 1; Table 1). Trophic chain length (i.e. maximum/highest TP per survey time; TCL) increased over time mostly likely due to an increase in taxa richness (Fig. 1; Table 2).

**Table 1:** Invertebrate taxa sampled from the ephemeral pond over the course of a hydroperiod used for stable isotope analyses. Abbreviations: Taxa abbr. – taxa abbreviation (i.e. assigned sample number), *n* – number of analysed samples, TP – trophic position.

Taxa	Taxa abbr.	Survey 1			Survey 2			Survey 3			Survey 4		
		*n*	δ^13^C (‰)	TP	*n*	δ^13^C (‰)	TP	*n*	δ^13^C (‰)	TP	*n*	δ^13^C (‰)	TP
**Macroinvertebrate**													
*Aeshna* sp.	17				6	−24.8 (0.9)	2.4 (0.1)	4	−27.1 (0.3)	1.7 (0.1)	5	−26.1 (0.3)	4.3 (0.4)
*Anisops* sp.	18				4	−26.0 (4.1)	2.1 (0.2)	8	−25.6 (2.6)	2.7 (0.7)			
*Bulinus tropicus*	19				6	−13.0 (1.7)	1.5 (0.2)	7	−19.1 (1.5)	1.2 (0.1)			
*Baetis* sp.	30							3	−30.1 (0.02)	2.2 (0.01)			
*Diplonychus capensis*	31							4	−25.5 (0.4)	2.5 (0.2)			
*Chironomus* sp.	32							7	−24.6 (1.2)	2.2 (0.1)			
Chlorolestidae	20				2	−31.1 (0.02)	1.5 (0.01)						
*Cypricercus* sp. 1	35										2	−21.1 (0.7)	1.3 (0.3)
*Cypricercus* sp. 2	1	5	−18.9 (0.3)	1.6 (0.1)							3	−21.4 (1.8)	1.5 (0.3)
*Cyzicus* sp. 1	2	4	−22.9 (0.4)	1.7 (0.1)	6	−21.9 (3.3)	1.6 (0.2)	5	−19.6 (2.4)	2.6 (0.4)	2	−17.6 (0.1)	2.4 (0.1)
*Cyzicus* sp. 2	36										5	−26.1 (0.6)	1.1 (0.3)
*Enithares sobria*	21				6	−26.9 (1.1)	2.8 (0.5)	3	−25.6 (0.02)	3.6 (0.1)			
Elmidae	22				3	−24.0 (1.7)	2.7 (0.6)	4	−23.4 (1.9)	1.6 (1.0)			
Hirudinae	23				2	−20.2 (0.3)	2.7 (0.3)	3	−23.3 (1.4)	2.6 (0.4)	2	−21.2 (0.8)	2.3 (0.1)
Hydracarina	33							2	−28.4 (0.3)	2.7 (0.01)			
*Lynceus* sp. 1	24				2	−24.0 (0.4)	1.7 (0.1)	7	−24.4 (0.7)	1.3 (0.2)	5	−23.7 (0.5)	1.5 (0.3)
*Sigara* sp.	25				2	−22.4 (0.01)	2.4 (0.02)	7	−22.7 (0.4)	2.5 (0.2)			
*Streptocephalus* sp.	3	2	−23.5 (0.02)	1.3 (0.01)	6	−27.3 (1.2)	2.0 (0.1)				2	−21.2 (0.01)	1.9 (0.01)
*Triops granarius*	4	2	−22.5 (0.03)	1.8 (0.01)	5	−22.6 (1.5)	2.2 (0.3)						
**Zooplankton**													
*Daphnia longispina*	5	3	−23.0 (0.2)	1.7 (0.03)	4								
*Daphnia magna*	6	4	−23.3 (0.4)	2.2 (0.7)	6	−27.8 (0.5)	1.8 (0.5)	6	−28.1 (0.3)	1.9 (0.1)			
*D. longispina* + *Kurzia* spp.	26				7	−29.2 (0.5)	1.6 (0.1)	4	−27.7 (0.8)	1.7 (0.04)	5	−26.7 (0.1)	1.6 (0.2)
*Lovenula raynerae*	7	8	−21.4 (.0.8)	2.5 (0.3)	17	−23.6 (0.8)	3.1 (0.1)	16	−24.0 (0.6)	3.1 (0.3)	17	−25.2 (1.0)	2.8 (0.2)
*Paradiaptomus lamellatus*	8	7	−21.6 (0.7)	2.0 (0.3)	17	−26.1 (0.9)	2.0 (0.2)	8	−24.7 (0.5)	1.7 (0.4)	17	−25.3 (0.9)	2.7 (0.5)
*Mesocyclops* sp. + nauplii	9	3	−23.1 (0.8)	2.1 (0.2)	4	−23.9 (0.5)	2.9 (0.4)	4	−23.9 (0.6)	2.4 (0.1)	4	−23.9 (0.8)	2.5 (0.2)

**Table 2:** Community-wide metrics calculated using SIBER analyses based on the invertebrate taxa δ^13^C and trophic position values. Abbreviations: dCr – carbon range, TA – total convex hull area, MNND – mean nearest neighbour distance values, SEAc – standard ellipse areas, TP – trophic position (measure of trophic chain length).

Survey	dCr	TA	MNND	SEAc	TP
1	5.2 (5.1–5.3)	8.1 (7.7–8.5)	0.13 (0.11–0.15)	3.3 (3.0–3.6)	2.5
2	19.3 (18.8–20.3)	25.4 (24.4–26.9)	0.14 (0.12–0.15)	7.3 (6.7–7.9)	3.1
3	12.9 (12.6–13.8)	27.6 (25.7–29.8)	0.13 (0.12–0.15)	5.9 (5.5–6.3)	3.6
4	9.0 (9.2–9.4)	19.6 (19.0–21.3)	0.14 (0.12–0.17)	4.9 (4.3–5.4)	4.3

**Figure 2:**
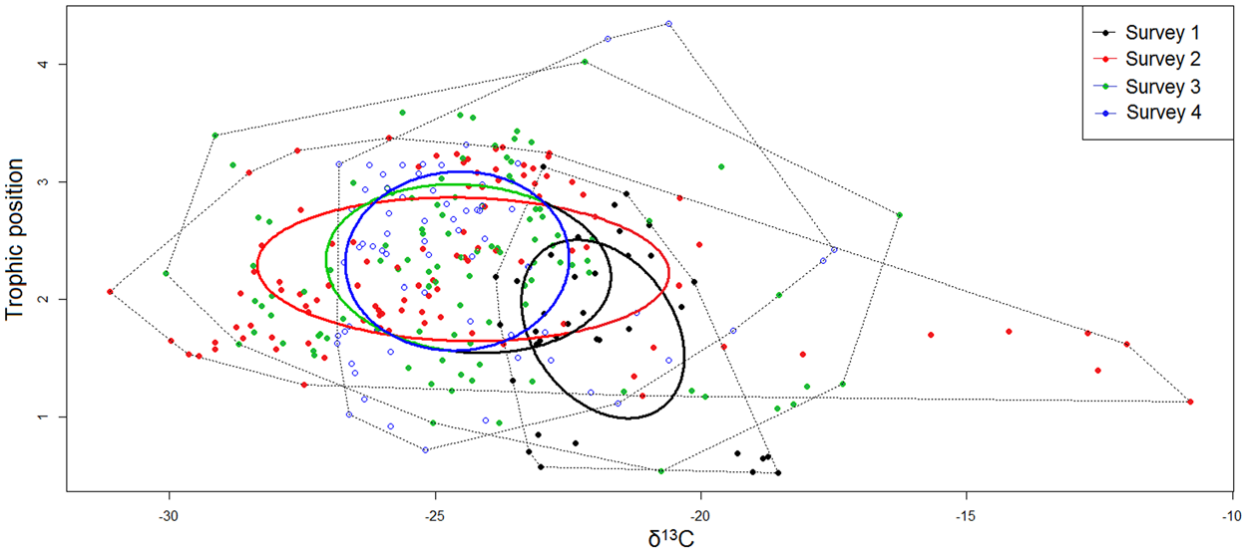
Trophic diversity for invertebrates over the course of a hydroperiod for an ephemeral pond depicted by total convex hull areas (dotted lines) and standard Bayesian ellipse areas (solid lines).

Taking the entire trophic structure as a whole, the overall mean consumer δ^13^C carbon range (dCr) was wider during survey 2 compared to all other surveys (Figs 1 and 2; Table 2), with the dCr range showing a trend similar to the mean core isotopic niche width (SEAc; Table 2). All surveys had a trapezoid area shape foodweb, with survey 1 being narrow along both axes and survey 2 being wider along the δ^13^C axis with an increase in the trophic web chain length (TP – survey 2 = 3.1 *vs* TP – survey 1 = 2.5; Figs 1 and 2; Table 1). During survey 3, however, the δ^13^C axis became narrower while the foodweb chain increased to 3.6 (Figs 1 and 2; Table 1). During survey 4, the food web narrowed further along the δ^13^C axis while the TP axis increased marginally to 4.3. The Layman metric SEAc also captured the differences in trophic niche space between the surveys. This procedure highlighted the trophic position and δ^13^C axes changes over time with a larger area towards the δ^13^C axis being occupied during survey 2 (Fig. 2, Table 2). The total trophic isotopic area (TA) and SEAc were significantly different between the hydroperiods, with the mean nearest neighbour distance (MNND) showing no variation (Table 2).

There were significant and negative correlations for TCL vs water depth (as a proxy of hydroperiod/survey time; *r*_4_ = −0.98, *P* = 0.02) and positive correlation for taxa richness vs TA (*r*_4_ = 0.97, *P* = 0.02). Non-significant and high correlations were observed for TCL *vs* TA (*r*_4_ = 0.51, *P* = 0.47), water depth (as a proxy of hydroperiod/survey time) *vs* TA (*r*_4_ = −0.67, *P* = 0.33), taxa richness *vs* dCr (*r*_4_ = 0.85, *P* = 0.14) and SEAc (*r*_4_ = 0.91, *P* = 0.09).

## Discussion

Our results support the first and third hypothesis for the study, whereby the early stage food web was the shortest and simplest and food web length increased over time in response to insect predator arrival. In contrast to our second hypothesis, however, lower and intermediate trophic level structure evolved over time and isotopic variation increased, particularly with regard to carbon signatures. As such, temporal variation in δ^13^C was greater than that of δ^15^N. This change was likely an aspect of increases in diversity at lower and intermediate levels in relation to internal recruitment, in combination with feeding specialisation by key biota, such as the copepods *P. lamellatus* and *L. raynerae* as was highlighted by Dalu *et al*.^28^.

Results of the current study indicate that the dCr values varied during each survey suggesting differential use of carbon sources by the macroinvertebrates within the system over time (Fig. 1; Table 2). During the second survey, the carbon range of consumers was significantly broader, pointing to a combination of strategies where some taxa have a lower integration of carbon sources, while other co-occurring taxa integrate several carbon sources (Fig. 1; Table 2). In the pond ecosystems, much of the diversity was found to occupy the intermediate positions in the food web. In addition to the taxonomic diversity, much of the trophic diversity at this level was likely driven by both herbivory and omnivory. As demonstrated by the changes in trophic positions (between 1 and 3) for selected organisms, a more diverse diet that incorporates a higher amount of different food items was observed (see Fig. 1; Table 1). An enhanced feeding on lower trophic positions^29^ could potentially satisfy herbivore and omnivore energy demands during the different survey periods. This was likely supported by a greater diversity of primary producers (Fig. 1), which would have accumulated in the primary consumers that fed on them, thereby reducing their isotopic trophic position^29^. Omnivory therefore increases activity at intermediary levels, an important consideration when assessing trophic links in a food web. In addition, a higher functional redundancy in pond ecosystems was evidenced here by a closer mean nearest neighbour distance (MNND), indicating that more species occupied similar trophic web positions (Table 1).

The foodweb shapes as depicted by the SEAc, TA and other Layman community metrics differed among the survey times (Figs 1 and 2). In essence these results highlight that the trophic food web length in the ephemeral pond changes in response to water depth, a correlate of time (hydroperiod). While trophic chain length (TCL) and overall taxa richness increased over the duration of the investigation; the diversity of predators predicted to increase significantly over time was not observed. Results of the stable isotope study suggest that the system was characterised by multi-chain omnivory, with one top predator integrating the different carbon sources fuelling the trophic structure^30^. The trapezoid shape with a wider TP level 2 of the trophic structure suggests that much of the trophic complexity in the ephemeral pond is driven by midriff expansion rather than diversification at the top of the food web. This is likely facilitated through specialisation by certain contributors (omnivores) at the intermediary trophic levels^28^. Thus, some of the changes observed in food web shape could be a result of changes in the trophic position of the macroinvertebrates across the surveys.

**Figure 3:**
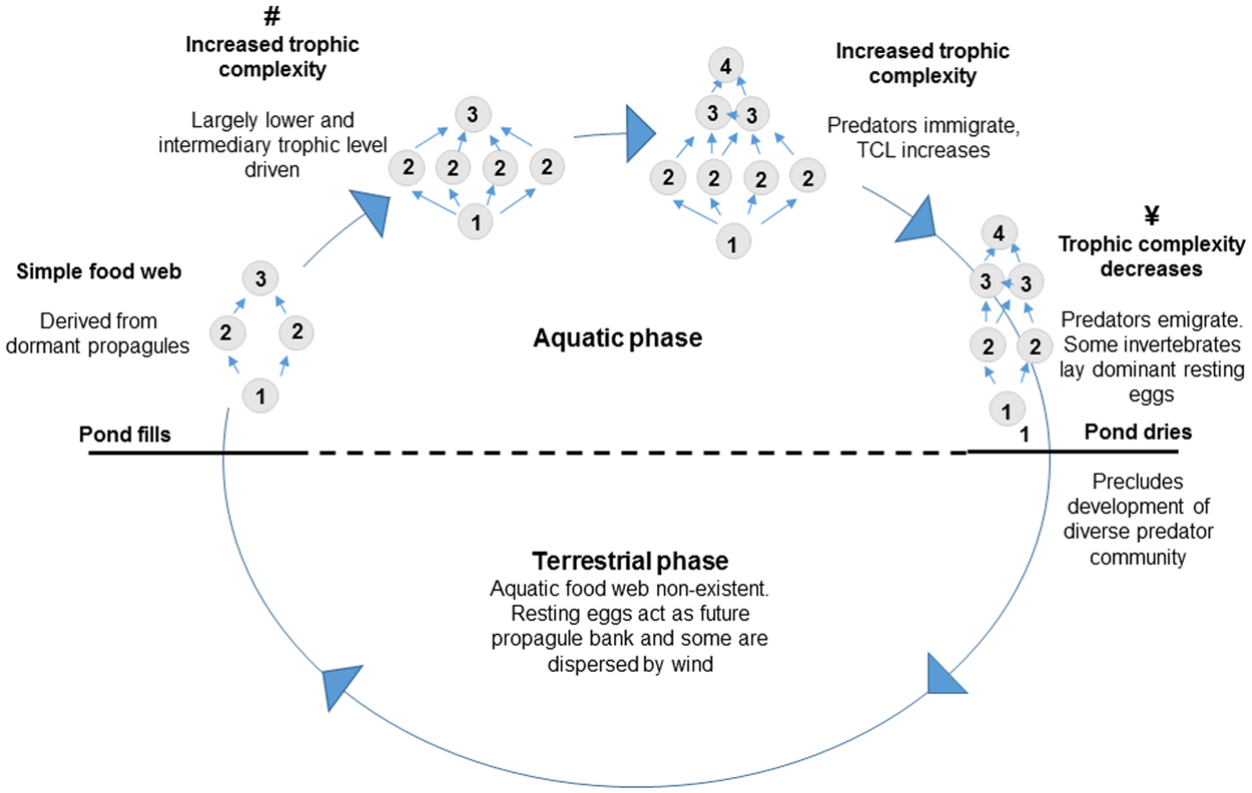
Proposed conceptual model of temporal food web changes outlining (**#**) intermediate trophic level widening due to increased richness, herbivory and/or omnivory, and (**¥**) mechanism reducing relative predator diversity increases. The model shows widening of the carbon range and elongation of the trophic structure with time before it becomes simple again. TCL = trophic change length.

Our conceptual model (Fig. 3), outlines the patterns of trophic structure change over time in the system. It is likely representative of foodweb dynamics in temporary aquatic ecosystems of a similar climate. However, it is important to highlight that the model might differ from one region to another, particularly with regard to differences in hydroperiod associated with local rainfall and evaporation processes.

The use of δ^15^N isotopes for TCL estimation, does however, need to be interpreted with caution given that we employed a single average trophic fractionation value (i.e. 2.3‰ used in this study). Trophic fractionation values are unlikely to be constant throughout the entire foodweb^5, 31^. We assumed that there were no trophic enrichment differences during the different surveys as the main organism groups were found throughout the study, with sampling conducted at the same sites. Thus, we have no reason to suppose that the observed differences were a result of various factors other than the high average number of trophic steps during the different survey times. As such, these aspects do not take away from the main findings of the study within the context of the main hypotheses.

The study provides empirical evidence on the dynamic nature of food webs in an ephemeral ecosystem over a hydroperiod. The realised TCL estimated during this study are, generally lower than those recorded for organisms in permanent freshwater bodies^5, 32, 33^. Predator diversity in ephemeral ponds is heavily reliant on an immigration process from other environments and ultimately results in limited predator diversity levels when compared to permanent systems. Our findings were in agreement with those of O’Neill & Thorp^12^, O’Neill *et al*.^19^, Schalk *et al*.^34^ and Schriever & Williams^35^ on ephemeral ecosystems, whereby greater taxa richness contributed to increased trophic complexity over time. Given the temporary nature of the ponds, the largest predators are still small-bodied when compared to those that occupy the tops of food webs in permanent water bodies. Large predacious aquatic organisms such as fish and malacostraca are typically unable to survive in ephemeral wetlands and as such the amplitude of ephemeral pond food webs will never reach the levels observed in permanent bodies as isotopic nitrogen levels are limited by the drying out process. This has implications for food web shape as δ^15^N variations are intrinsically limited within these systems, while δ^13^C variations are not.

The dynamic nature of these systems seems to be conducive to greater levels of intermediate and lower trophic level diversity, with omnivorous traits likely being advantageous. When considering these findings within the broader context of aquatic environments, the question of predator diversity accumulation as a function of environmental stability arises. Pelagic marine environments are arguably the most stable of aquatic ecosystems^36^ which likely facilitates the high diversity of predators, while ephemeral ponds are arguably the least stable. As such, future investigations should address changes in trophic chain length and shape at large multi spatio-temporal scales and across a range of aquatic ecosystems, from impermanent freshwaters through to the most permanent of large freshwater and marine bodies, representing a gradient of habitat stability.

## Materials and Methods

### Study site

The study was conducted in an ephemeral pond situated at Burnt Kraal (33′15 °S, 26′26 °E) in the south–eastern temperate region of South Africa, over the period 24 June to 23 September 2015. When full, the pond had a surface area of 1952 m^2^ (0.48 acres) and mean depth of 0.38 ± 0.02 m (*n* = 3, measured using a graduated measuring rod). The mean summer and winter daily temperatures and annual rainfall for the area are 20.3 °C, 12.3 °C and 680 mm, respectively^37^. The first sampling event took place two weeks after a high rainfall (>100 mm) event that rapidly filled the dry pond, with the last sampling occurring two weeks before the pond completely dried out (Fig. 1). Sampling was conducted at four discrete periods over the course of a pond’s hydroperiod: survey 1 (24 June 2015: mean depth at deepest point 0.38 ± 0.02 m), survey 2 (27 July 2015: mean depth 0.24 ± 0.03 m), survey 3 (29 August 2015: mean depth 0.16 ± 0.01 m) and survey 4 (23 September 2015: mean depth 0.09 ± 0.01 m).

### Sample collection and processing

Stable isotope samples for invertebrates and plants were collected on each sampling period at midday (Table 1). No vertebrates were encountered while sampling the ephemeral pond. Zooplankton samples (*n* = 4–17) were collected by towing horizontally a zooplankton net (63 µm mesh size, 32 cm diameter) through the water column. Macroinvertebrate larval samples (*n* = 2–8) were also collected using a nylon hand net (500 μm mesh size, 30 × 30 cm dimension). All zooplankton and macroinvertebrates were identified to the lowest taxonomic resolution using keys by Day *et al*.^38, 39^, Fernando^40^, Gerber & Gabriel^41^, Gooderham & Tsyrlin^42^ and Suárez–Morales *et al*.^43^. All samples were then stored in toluene-cleaned labelled Eppendorf tubes for later processing in the laboratory.

Detritus (*n* = 4), horse dung (*n* = 4), macrophyte leaves (*n* = 4) and terrestrial grass (*n* = 3–5) were collected on each sampling trip by hand and placed in labelled zip-lock bags. Four surface water samples ~20 cm depth were collected in 20 L carboys for the determination of particulate organic matter (POM). The POM water was then further filtered through pre–combusted (450 °C, 5 h) Whatman GF/F filters after initial filtering through a 63 µm mesh to remove zooplankton. Any visible zooplankton on the filters, that might have escaped pre-screening were removed with forceps under a dissecting Olympus microscope operated at 100 × magnification before being placed in separate labelled pre–combusted (450 °C, 5 h) aluminium foil envelopes. Sediment (*n* = 4) was collected using a van Veen grab (bite depth ~1–5 cm) and placed into sterile plastic bags. In the laboratory, all samples in zip-lock were placed into separate, labelled pre–combusted aluminium foil envelopes.

All plant material (detritus, macrophytes, grass, POM), dung, sediment and invertebrate samples were oven dried at 60 °C for 72 hrs before being further ground to a fine homogeneous powder using a mortar and pestle. To obtain sufficient amounts of material to conduct the stable isotope analysis, some zooplankton taxa were pooled together i.e. Cladocera (*Daphnia longispina* and *Kurzia* spp.) and Copepoda (*Mesocyclops* spp. and nauplii). Before placement into toluene cleaned tin capsules, dried sediment samples were acidified by vortexing for 2 min in 2 M hydrochloric acid, centrifuged at 3600 rpm for 5 min, washed in deionised water twice followed by re-centrifugation, drying at 50 °C and homogenising in a Retsch Mixer Mill^26^. Aliquots of approximately 0.6–0.7 mg (animals) and 1–1.2 mg (plant material, dung, sediment) were weighed into aluminium tin capsules that were pre–cleaned in toluene.

### Stable isotope sample analysis

Stable isotope analysis was carried out using a Flash EA 1112 Series coupled to a Delta V Plus stable light isotope ratio mass spectrometer via a ConFlo IV system (Thermo Fischer, Bremen, Germany), housed at the Stable Isotope Laboratory, University of Pretoria. Merck Gel (δ^13^C = −20.57‰, δ^15^N = 6.8‰, C% = 43.83, N% = 14.64) standards and blank sample were run after every 12 unknown samples. All results were referenced to Vienna Pee–Dee Belemnite and to air for carbon and nitrogen isotope values, respectively. Results were expressed in delta notation using a per mille scale from the standard equation 1:

1 $$\delta X\,(\textperthousand)=[\frac{{\rm{R}}{\rm{s}}{\rm{a}}{\rm{m}}{\rm{p}}{\rm{l}}{\rm{e}}}{{\rm{R}}{\rm{s}}{\rm{t}}{\rm{a}}{\rm{n}}{\rm{d}}{\rm{a}}{\rm{r}}{\rm{d}}}-1]\times 1000$$

where X = ^15^N or ^13^C and R represents ^15^N/^14^N or ^13^C/^12^C, respectively. Average analytical precision was <0.15‰ for δ^13^C and <0.1‰ for δ^15^N.

The trophic positions of all invertebrates in the pond were estimated using the formula of Vander Zanden^44^ (equation 2):

2 $${\rm{Trophic}}\,{\rm{position}}\,({\rm{TP}})=2+\frac{{}^{15}{\rm{N}}_{{\rm{consumer}}}-{}^{15}{\rm{N}}_{{\rm{zooplankton}}}}{2.3}$$

where: δ^15^N_consumer_ is the measured consumer δ^15^N for which TP needs to be estimated and δ^15^N_zooplankton_ is the average δ^15^N of the primary consumer (mean of Cladocerans) at a particular hydroperiod and 2.3 is the trophic fractionation for δ^15^N^45^. The level 2 was consequently attributed, empirically, to zooplankton i.e. Cladocera^26^.

### Data analysis

To investigate the ephemeral pond trophic structure, quantitative population metrics^27^ were derived for each species using the Stable Isotope Bayesian Ellipses (SIBER)^46^ model in R. Despite the fact that the estimation of these metrics is usually made using raw δ^15^N^27^, they have also been estimated by standardising δ^15^N to trophic chain length (i.e. trophic position). We used the latter method in our study as it shows reduced variability in δ^15^N due to factors other than trophic fractionation^5, 47^. Layman metrics included the mean distance to the centroid (CD), which provides a description of trophic diversity (niche width and species spacing); dCr (δ^13^C range), providing a univariate estimate as a measure of the diversity of basal resources; mean nearest neighbour distance (MNND), providing a measure of density and clustering of species within the community and standard ellipse area (SEAc), which provides a bivariate measure of mean core isotopic niche width (see Jackson *et al*.^46^ for detailed methodology). To allow comparisons between species populations with varying sample sizes, all metrics were bootstrapped (*n* = 9999) and a small sample size correction for improving accuracy to SEA values is indicated by the subscript ‘c’^46^. Pearson correlations were then carried out to assess the relationships between trophic chain length (TCL; i.e. maximum/highest TP value per survey time) *vs* invertebrate taxa richness, dCr, TA and water depth (as a proxy of hydroperiod).

### Data Accessibility

Once the paper has been accepted for publication, a data manuscript will be finalised and submitted to Scientific Data.
